# Relationship between screen time among children and lower economic status during elementary school closures due to the coronavirus disease 2019 pandemic

**DOI:** 10.1186/s12889-022-12559-5

**Published:** 2022-01-24

**Authors:** Sangha Lee, Sungju Kim, Sooyeon Suh, Hyojin Han, Jaeoh Jung, Sujin Yang, Yunmi Shin

**Affiliations:** 1 Department of Psychiatry, Ajou University School of Medicine, Suwon, Republic of Korea; 2 Department of Psychology, Sungshin Women’s University, Seoul, Republic of Korea; 3 Institute of future convergence, Yonsei University, Seoul, Republic of Korea

**Keywords:** Screen time, COVID-19, School closure, Lower income

## Abstract

**Background:**

This study aimed to examine whether the extended use of a variety of digital screen devices was associated with lower economic status and other environmental factors among Korean elementary school children and their caregivers during school closures precipitated by the coronavirus disease 2019 (COVID-19) pandemic.

**Methods:**

A total of 217 caregivers of children 7–12 years of age from Suwon, Korea, were recruited and asked to respond to a self-administered questionnaire in June 2020. The questionnaire addressed demographic information and children’s use of digital media, in addition to their caregivers. The *t*-test was used for continuous variables, and the Kruskal-Wallis test was used for variables measured on an interval scale. A multiple regression analyses were performed to examine the effects of significant correlative factors on screen time in children as predictors.

**Results:**

Children with lower household incomes demonstrated a higher frequency and longer duration of smartphone and tablet personal computer use compared to those from higher income households. Children of households in which incomes decreased after COVID-19 used smartphones and tablet PCs more often and for longer durations. Children from households that experienced decreased income(s) after COVID-19 used personal computers more often and for a longer duration, and children from low-income families engaged in longer screen time on smartphones. A change in primary caregiver(s) may have increased children’s screen time on smartphones.

**Conclusion:**

Lower household income was associated with longer screen time among children, and poor mental health among caregivers during school closures precipitated the COVID-19 pandemic.

## Background

Social distancing policies, precipitated by coronavirus disease 2019 (COVID-19), which has spread worldwide since early 2020, have dramatically changed lives. The COVID-19 outbreak has resulted in school closures, which have forced predominantly online education for children. As of April 8, 2020, schools were closed in 188 countries according to the United Nations Educational, Scientific and Cultural Organization (i.e., UNESCO) [1]. As a result, students spend more time at home, which has resulted in inevitable changes in screen time among elementary school students [2].

Previous studies investigating screen time among toddlers and children have been actively conducted over the past decade and have revealed an exponential increase in multimedia use. Various researchers have described the effects of prolonged screen time on children, and guidelines for screen time according to age have been published. For example, the World Health Organization has suggested that sedentary screen time should not exceed 1 h per day and, moreover, the less the better. Despite such guidelines, screen time among infants, preschoolers, and school-age children has been steadily increasing annually, especially for mobile devices such as smartphones and tablet personal computers (PCs) [3].

COVID-19 has also had a tremendous impact on economies, with businesses providing interpersonal services facing unprecedented difficulties. The World Labor Organization estimates that the number of unemployed individuals worldwide will increase by up to 24 million due to COVID-19 [4]. In this economic crisis, specific groups may be more vulnerable than others to the psychosocial effects of the pandemic. Previous studies have reported that parents’ economic status is significantly associated with their children’s screen time [5–8]. A study reported that highly engaged screen use is more likely among children with low socioeconomic status (SES) than among those with higher SES [9]. Thus, it is plausible that COVID-19 may have had differential effects on screen use according to household income. According to a nationally representative survey from the United States, caregivers reported significant stress with financial difficulty in more than one-third of households with young children, and 63% reported that they have lost emotional support during the pandemic [10]. More specifically, caregivers’ mental health can also affect children’s social impairment and depression, which is also related to media use by children. Maternal depression was associated with children’s television (TV) overuse in a previous study conducted in South Korea before COVID-19, but no significant results were found among other devices [11]. Another study involving 1098 parents and children in Hong Kong reported a positive correlation between parental depression and adolescent Internet addiction [12].

However, there is little information about the impact of COVID-19 on children living in households that experienced a marked decrease in household income due to COVID-19. As such, the specific effects of school closures on children and their caregivers’ mental health are, in large part, unknown. Thus, this study aimed to investigate the differential effects of low SES and the impact it has had on children’s screen time and their caregivers’ psychological health, with a focus on household income levels and changes in income since the onset of the COVID-19 pandemic.

## Methods

### Participants

The present study was conducted in June 2020 to investigate various screen-device usage patterns of various media (TV, tablet PC, and smartphones) during the period of school closures due to COVID-19, and to identify psychosocial factors that had a significant impact on children’s screen time and behavioral problems. In South Korea, classes for K-12 students were replaced with online-only classes from March to May 2020. In the case of elementary schools, in-person classes have been reduced from five days per week to one to three days per week since June 2020. The data presented in this article include those collected from March to May 2020, an approximately three-month period during which South Korea’s online-only school curriculum was implemented.

A total of 217 caregivers of elementary school children were recruited from community centers in Suwon, Gyeonggi-do, South Korea. Interested parents who contacted the study team were e-mailed a link to the questionnaire. The criterion for participant enrollment was having elementary school or preschool children; the authors verified whether they owned each device. Children with serious developmental disabilities, such as autism or intellectual disability, were excluded from the study. The study was approved by the institutional review board (IRB) of the Ajou University School of Medicine, Suwon City, South Korea (AJIRB-SBR-SUR-14-378). Informed consent was obtained from all participants. Participants who consented to participate in the study and fulfilled the inclusion criteria, and were able to complete the survey set, were compensated for their time ($5 worth of masks and hand sanitizer).

### Measures

#### Body mass index

All respondents were asked to provide their children’s height and weight. Based on this information, the researchers calculated the body mass index (BMI; kg/m^2^)*.*

#### Household income

Household income was assessed using the question, “What is your average monthly household income?” Caregivers answered according to two response options: “less than $3400 (4,000,000 Korean won, 4M KRW)”; and “over $3400 (4,000,000 KRW)”. Normally, the Korean government subdivides the range of household income into 10 for the purpose of using it in support of policies for low-income families, which is calculated in association with the median national income. In this study, the income section was divided based on 4 million won (approximately $3400 USD) by referring to this guideline. The changes in household income after COVID-19 were assessed using the question “Has your household income changed since COVID-19?” Caregivers responded according to three options: “not changed,” “increased,” or “decreased”.

#### Screen time

Primary daytime caregivers were queried about their personal screen time. Questions addressed the frequency and duration of use among children regarding digital screen devices (i.e., TVs, tablet PCs, smartphones) in the past 3 months or when the school was closed. In response to the frequency of use, a request was made to enter the number of days each device was used for 1 week; the response options for the mean length of time per day using media and corresponding scores were as follows: none (score, 0); < 1 h (score, 1); 1–2 h (score, 2); > 2–3 h (score, 3); > 3–4 h (score, 4); and ≥ 4 h (score, 5).

#### Subjective stress among caregivers

Caregiver subjective stress was assessed using the question “Please write the number that best describes your level of stress over the past week and scored as “If it is very difficult, it is 10 points, and if it is not difficult at all it is 0.”

#### Caregiver depression

Caregiver depression levels were assessed using the Patient Health Questionnaire-9 (PHQ-9). The PHQ-9 is a self-report measure that asks whether the subject had experienced the following problems in the past 2 weeks: little pleasure or interest in doing things; feeling down, depressed, or hopeless; sleeping too little or too much; feeling tired or having little energy; poor appetite or overeating; feelings of worthlessness or guilt; concentration problems; psychomotor impairment or agitation; and thoughts of suicide (“Thoughts that you would be better off dead or of hurting yourself in some way”). Subjects were asked to rate how often each symptom occurred, as follows: 0 (not at all), 1 (several days), 2 (more than half the days), or 3 (nearly every day). The total PHQ-9 score for the nine items ranged from 0 to 27, with higher scores indicating more depressive symptoms. The Cronbach’s α in this study was 0.90.

### Statistical analyses

Data were analyzed using SPSS version 25.0 (IBM Corporation, Armonk, NY, USA). Frequency analysis and descriptive statistics were performed for demographic data. For data regarding continuous variables, a *t*-test or multiple regression analysis was used, and the Kruskal-Wallis test was used for data composed of the sequence scale. To control variables that could affect children’s screen time, a correlation analysis between each variable was performed, followed by linear multiple regression analysis.

## Results

### Participant demographics

Demographic data are summarized in Table 1. The mean (± standard deviation) age of the children was 9.16 ± 1.43 years, and the average BMI was 18.14 ± 3.3 kg/m^2^. Mothers were the primary caregivers in most cases (89.4%), and 20.3% reported that their main caregivers had changed after COVID-19. Household income was divided into two levels, with 53.9% of respondents reporting a monthly income < $3400 USD, and 46.1% earning > $3400 USD per month.

**Table 1:** Demographic characteristics of the study participants (*n* = 217)

Variables	Mean ± Standard Deviation
	Number (%)
Sex, female	95 (43.8%)
Age	9.16 ± 1.43
Body Mass Index (BMI)	18.14 ± 3.30
Caregiver	
Mother	194 (89.4%)
others	23 (10.6%)
Not changed	173 (79.7%)
Changed	44 (20.3%)
Monthly household income	
Less than $3400 (USD)	117 (53.9%)
$3400 (USD) or more	100 (46.1%)
Monthly household income change Since COVID-19 outbreak	
decreased	67 (30.9%)
not changed	144 (66.4%)
increased	6 (2.8%)

### Results based on household income levels and changes

Table 2 summarizes the differences in mean digital device use frequency, screen time, caregiver stress, and PHQ score compared according to income status and groups in which the caregiver changed. There were significant differences between the low-income and above-average income groups in smartphone use frequency per week and daily screen time. The low-income group exhibited a significantly higher frequency of smartphone use compared to the above-average income group (5.05 ± 2.68 h versus [vs.] 3.97 ± 2.68 h, respectively; *p* < 0.0033). The low-income group also reported significantly higher daily smartphone use time than the above-average income groups (3.52 ± 1.64 vs. 2.60 ± 1.36, respectively; *p* < 0.0167). The group with decreased income status since the COVID-19 outbreak demonstrated significantly higher usage frequencies per week of tablet PC (4.30 ± 2.68 vs. 3.14 ± 2.82, respectively; *p* < 0.0167) and smartphone (5.27 ± 2.52 vs. 4.23 ± 2.89, respectively; *p* < 0.0167) than the non-decreased income status group.

**Table 2:** Children’s screen time variables and caregiver’s mental health compared by household income groups and caregiver changes

	All participants	Low-income level	Above-average level	*p*-value	Decreased income status	Not-decreased income status	*p*-value	Caregiver changed	Caregiver unchanged	*p*-value
	*N* = 217 Mean ± SD	*N* = 117 Mean ± SD	*N* = 100 Mean ± SD		*N* = 67 Mean ± SD	*N* = 150 Mean ± SD		*N* = 44 Mean ± SD	*N* = 173 Mean ± SD	
Children’s variables										
TV frequency per week^a^	4.79 (2.46)	4.87 (2.56)	4.69 (2.35)	0.589	4.67 (2.79)	4.84 (2.31)	0.666	5.41 (2.29)	4.63 (2.49)	0.061
TV screen time per day^b^	3.27 (1.30)	3.49 (1.47)	3.02 (1.03)	0.017	3.27 (1.40)	3.27 (1.26)	0.822	3.14 (1.19)	3.31 (1.33)	0.397
Tablet PC frequency per week^a^	3.50 (2.87)	3.92 (2.98)	3.00 (2.66)	0.017	4.30 (2.83)	3.14 (2.82)	0.006*	3.23 (2.97)	3.57 (2.85)	0.485
Tablet PC screen time per day^b^	2.76 (1.62)	3.04 (1.72)	2.43 (1.42)	0.010*	3.36 (1.74)	2.49 (1.49)	0.001**	2.84 (1.92)	2.74 (1.54)	0.921
Smartphone frequency per week^a^	4.55 (2.82)	5.05 (2.68)	3.97 (2.88)	0.005**	5.27 (2.52)	4.23 (2.89)	0.009*	5.64 (2.21)	4.28 (2.89)	0.001**
Smartphone screen time per day^b^	3.10 (1.58)	3.52 (2.60)	1.64 (1.36)	0.000**	3.81 (1.72)	2.78 (1.41)	0.000**	3.57 (1.59)	2.98 (1.56)	0.031
Caregiver’s variables										
Subjective stress scores^a^	5.49 (2.56)	5.93 (2.64)	4.98 (2.37)	0.006*	5.97 (2.84)	5.28 (2.40)	0.066	6.36 (2.39)	5.27 (2.58)	0.011*
Patient Health Questionnaire −9 ^a^	5.33 (5.55)	6.94 (6.39)	3.45 (3.57)	0.000**	6.60 (5.17)	4.77 (5.64)	0.024	7.20 (6.49)	4.86 (5.20)	0.012*

^a^ t-test

^b^ Kruskal-Wallis test

* significant at *p* < .0167 levels

** significant at *p* < .0033 levels

Caregivers’ PHQ score was significantly higher (*p* < 0.0167) in the group with caregiver change (i.e., children whose primary daytime caregiver changed during school closure), with a mean of 7.20 ± 6.49 compared with the caregiver unchanged group, with 4.86 ± 5.20. Caregivers’ subjective stress level was also higher in the caregiver changed group (6.36 ± 2.39 vs. 5.27 ± 2.58, respectively; *p* < 0.0167). The frequency of smartphone usage per week was significantly higher in the caregiver changed group compared to the caregiver unchanged group (5.64 ± 2.21 vs. 4.28 ± 2.89, respectively; *p* < 0.0033). Other children’s screen time variables were not significantly different between the caregiver changed and unchanged groups.

### Results of linear multiple regression analysis

Table 3 summarizes correlations between children’s screen time variables and the associated factors of children and their caregivers. Low household income level was weakly correlated with usage frequency per week of tablet PC (*r* = 0.161, *p* = 0.018) and smartphone use (*r* = 0.192, *p* = 0.005). The daily screen time for all digital devices (TV, *r* = 0.179, *p* = 0.008; tablet PC, *r* = 0.190, *p* = 0.005; smartphone, *r* = 0.291, *p* < 0.0001) was correlated with a low household income level. Decreased household income since the COVID-19 outbreak was significantly correlated with the usage frequency per week of tablet PC (*r* = 0.187, *p* = 0.006) and smartphone use (*r* = 0.170, *p* = 0.012). The daily screen time for tablet PCs and smartphones was correlated with decreased household income (tablet PC, *r* = 0.248, *p* < 0.0001; smartphone, *r* = 0.300, *p* < 0.0001).

**Table 3:** Correlations of children’s screen time variables and participants’ associated variables

	Household income variables		Children’s variables		Caregivers’ variables			
	Low-income	Decreased income	Sex	Body Mass Index	Mother as caregivers	Unchanged caregivers	Subjective stress	Depressive scores
TV frequency per week	0.037	−0.032	− 0.069	0.048	− 0.066	− 0.127	0.125	0.092
TV screen time per day	0.179^**^	− 0.002	− 0.001	0.131	− 0.032	0.053	− 0.002	0.153^*^
Tablet PC frequency per week	0.161^*^	0.187^**^	−0.058	0.085	−0.055	0.048	0.101	0.123
Tablet PC screen time per day	0.190^**^	0.248^**^	0.019	0.161^*^	0.014	−0.025	0.171^*^	0.159^*^
Smartphone frequency per week	0.192^**^	0.170^*^	0.028	0.129	0.009	−.194^**^	0.107	0.081
Smartphone screen time per day	0.291^**^	0.300^**^	0.036	0.172^*^	0.040	−.151^*^	0.127	0.062

* significant at *p* < .05 levels

** significant at *p* < .01 level

Having the mother as the main caregiver did not correlate with screen time for all devices; however, whether the main caregiver remained unchanged after school closure demonstrated a significant negative correlation with weekly frequency and daily screen time for smartphone use (*r* = − 0.194, *p* = 0.004, *r* = − 0.151, *p* = 0.027, respectively). Stress levels of the caregiver demonstrated a weak correlation with daily screen time for tablet PC (*r* = 0.171, *p* = 0.012), and the depression level of the caregiver demonstrated a weak but significant correlation with the screen time for TV and tablet PC (*r* = 0.153, *p* = 0.024; *r* = 0.159, *p* = 0.019, respectively).

Multiple linear regression analyses were performed to examine the effects of significant correlative factors on screen time in children as predictors (Table 4). The models explaining TV frequency (R^2^ = 0.085, *p* < 0.001), TV usage time (R^2^ = 0.066, *p* < 0.001), tablet usage time (R^2^ = 1.908, *p* < 0.001), smartphone frequency (R^2^ = 4.157, *p* < 0.001), and smartphone usage time (R^2^ = 3.718, *p* < 0.001) were found to be statistically significant (Table 4); however, the model for tablet PC frequency was not. Based on this result, children’s BMI was reported to be positively and significantly correlated with TV frequency and usage time (*β =* 0.176, *p* < 0.05; *β =* 0.196, *p* < 0.05, respectively). Interestingly, children’s age was reported to be negatively correlated with TV frequency and time (*β =* − 0.329, *p* < 0.01; − 0.202, *p* < 0.01, respectively), in contrast to positive correlations with smartphone frequency and usage time (*β =* 0.205, *p* < 0.01; − 0.299, *p* < 0.01, respectively).

**Table 4:** Multiple linear regression between screen time variables and associated factors

Model	Variables	B	SE	***β***	F(p)	Adj.R2
TV frequency per week	Child Factor					
	Age	−0.571	0.131	−0.329^**^	3.506^**^	0.085
	Sex	−0.300	0.328	−0.060		
	BMI	0.132	0.055	0.176^*^		
	Family factor					
	Low-monthly income	0.338	0.378	0.068		
	Decreased income	0.066	0.393	0.012		
	Caregiver factor					
	Not changed	−0.667	0.414	−0.108		
	Stress	0.082	0.071	0.086		
	Depression	0.025	0.034	0.056		
TV time per day	Child factor					
	Age	−0.185	0.070	−0.202^**^	2.896^**^	0.066
	Sex	−0.045	0.175	−0.017		
	BMI	0.077	0.029	0.196^*^		
	Family factor					
	Low-monthly income	0.451	0.202	0.173^*^		
	Decreased income					
	Caregiver factor	−0.072	0.210	− 0.025		
	Not changed	0.187	0.221	0.057		
	Stress	−0.047	0.038	− 0.093		
	Depression	0.044	0.018	0.187^*^		
Tablet frequency per week	Child factor					
	Age	0.107	0.157	0.053	1.908	0.033
	Sex	−0.516	0.391	−0.089		
	BMI	0.056	0.065	0.064		
	Family factor					
	Low-monthly income	0.282	0.451	0.049		
	Decreased income	0.889	0.469	0.144		
	Caregiver factor					
	Not changed	0.577	0.494	0.081		
	Stress	0.061	0.084	0.054		
	Depression	0.036	0.041	0.069		
Tablet time per day	Child Factor					
	Age	0.217	0.085	0.191^*^	4.157^**^	0.105
	Sex	−0.066	0.212	−0.020		
	BMI	0.042	0.035	0.085		
	Family factor					
	Low-monthly income	0.035	0.244	0.011		
	Decreased income	0.563	0.254	0.162^*^		
	Caregiver factor					
	Not changed	0.086	0.268	0.021		
	Stress	0.069	0.046	0.110		
	Depression	0.021	0.022	0.072		
Smartphone frequency per week	Child Factor					
	Age	0.408	0.149	0.205^**^	3.718^**^	0.092
	Sex	0.059	0.373	0.010		
	BMI	0.027	0.062	0.032		
	Family factor					
	Low-monthly income	0.604	0.430	0.107		
	Decreased income	0.305	0.448	0.050		
	Caregiver factor					
	Not changed	−1.195	0.472	−0.170		
	Stress	0.057	0.080	0.052		
	Depression	−0.013	0.039	−0.025		
Smartphone time per day	Child Factor					
	Age	0.333	0.079	0.299^**^	7.834^**^	0.203
	Sex	−0.031	0.197	−0.010		
	BMI	0.007	0.033	0.014		
	Family factor					
	Low-monthly income	0.502	0.226	0.158^*^		
	Decreased income	0.476	0.236	0.139		
	Caregiver factor					
	Not changed	−0.531	0.248	−0.134^*^		
	Stress	0.051	0.042	0.083		
	Depression	−0.028	0.021	−0.098		

*Adj.R*^*2*^ Adjusted R^2^

B = estimates

β = standardized beta

^*^ significant at *p* < .05 levels

^**^ significant at *p* < .01 levels

In terms of economic factors, children’s TV time and smartphone time increased when household monthly income was low (*β =* 0.173, *p* < 0.05; 0.158, *p* < 0.05, respectively), and the children’s tablet usage time increased in households whose income decreased to low after COVID-19 (*β =* 0.162, *p* < 0.05).

Children’s TV time increased as the level of depression of the main caregiver increased (*β =* 0.187, *p* < 0.05), and children whose main caregiver did not change after COVID-19 decreased their smartphone time (*β =* − 0.134, *p* < 0.05).

## Discussion

The present study compared children’s screen time and caregivers’ psychological health based on household income levels and changes in income in the new parenting environment created by COVID-19 and school closures. Unlike many previous studies, no distinction was made between screen time on weekdays and weekends because it was assumed that when schools closed, children’s time at home would no longer differ between weekdays and weekends. As expected, children in lower-income families were more likely to engage in longer digital screen times, and caregivers from lower-income families were more vulnerable to mental health problems.

The key findings and implications of this study are as follows. First, children from lower-income families and decreased-income families used screen devices more often and for longer periods, especially smartphones. This result is consistent with previous studies reporting that, even before COVID-19 [13], children from lower-income families engaged in longer screen times. On average, children from lower-income households spent > 1.5 h and sometimes close to ≥2 h per day with screen-based media. During school closures and social distancing, children’s screen time may have increased rapidly, which could exacerbate the risk for depression, anxiety, and inattention among children, especially those in lower-income families [14]. However, recent research investigating the mental health effects of children’s screen time suggests that screen time has a lower impact than other factors, such as parental support, family relationships, and childhood experiences [13]. Therefore, instead of simply reducing screen time, a more diversified support system may be needed for children of low-income households.

Second, caregivers of lower-income families reported higher levels of depression. Often, parents with low incomes are more vulnerable to stress and experience poor mental health. A meta-analysis of > 50 cross-national studies investigating the relationship between income and depression suggested that low-income individuals had a higher odds of depression [15]. Maternal depression is associated with negative parental behavior and difficulties with managing child behavior [16]. Depressed parents spend more time on smartphones or watching TV [17], and their screen time is positively correlated with that of their children [3]. In the current study, caregivers’ depression significantly explained 7.3% of children’s daily TV screen time. Although we could not identify possible mediating effects in other digital devices, based on these previous studies, we can explain the connection between caregivers’ depression and the high amount of screen time of children in lower-income families. Another possible explanation for the correlation between caregiver depression and a child’s long screen time may be caregiver guilt or frustration about the child’s long screen time(s). The American Academy of Pediatrics has recommended specific limits to screen time, < 1 h per day for 2–5-year-old children, although this is difficult to adhere to during COVID-19 [18]. A recent report from the United Nations Children’s Fund (i.e., UNICEF) suggested that although a small group of children will inevitably encounter adverse experiences related to digital technology, it is not directly related to the time spent online [19]. Mental health professionals and agencies need to revise their current guidelines addressing screen time to support caregivers who feel guilty about children’s excessive screen time during the COVID-19 pandemic.

The difference in screen time among children according to change(s) with the main caregiver(s) is also noteworthy. The parenting style of primary caregivers can affect children’s screen time in various aspects, including parenting practices, role modeling, parental perceptions of children’s screen viewing behaviors, parental self-efficacy, and general parenting style [20].

Along with school closures, some families have parents working from home (usually office workers), while in others, both parents must leave home to go to work due to a decrease in income. In this study, the screen time for each device according to changes in the primary caregiver was particularly noticeable with regard to smartphones. One possible explanation for this result is that when someone not familiar with using a smartphone, such as a grandparent, becomes the primary caregiver, control over smart devices may become more difficult. There is a significant difference between the parenting practices of fathers and mothers. In particular, maternal parenting practices have a significant impact on the screen time of children [21]. It is worth studying the changes in parenting style and structure caused by COVID-19 more closely and considering the education and support that can be provided to primary caregivers during the day.

Social distancing policies, such as school closures, are minimal measures for mitigating the spread of COVID-19; however, these measures are believed to affect mental health in the short and long term [22]. If the existing psychosocial support structure is weakened, a sense of isolation and vulnerability arises [23], which can lead to the overuse of media devices. In addition to the direct effect of COVID-19 mitigating the increase in children’s screen time, caregiver depression may also have an effect by mediating screen time. These additional effects can eventually create a vicious cycle of increasing screen time and worsening mental health. However, as noted above, reducing screen time is not the only factor—managing media content may be as important as limiting screen time. Many countries, including Korea, are replacing in-person classes with various online educational content during school closures. Although these factors increase screen time, they are unlikely to contribute to deterioration in mental health. However, even with short screen time(s), using content involving unethical, violent, and/or various trauma-related content can have serious adverse effects on children’s development. Therefore, accounting for the increase in time spent at home after COVID-19, it is necessary to provide more detailed content-usage guidelines for caregivers to prevent exploitation of their children by developmentally inappropriate media content.

Digital devices can be used not only in the educational field but also as a tool for improving social skills and providing interaction during periods of social distancing and isolation. Concerns regarding increased screen time due to COVID-19 have been accumulating; however, as Nagata et al. suggested, planning education, social support through social media, and even promotion of physical activity, can also be achieved through digital intervention [24]. A recent review found that using mobile applications or video games with physical activity components led to significant increases in physical activity among adolescents [25]. Another study suggested that during COVID-19, children familiar with social media may have an advantage in adapting themselves to the school-closure period and social distancing [26]. In our study, children’s BMI only significantly predicted the daily screen time for tablet PCs. Further research is needed to understand the relationship between screen time and physical status during the COVID-19 pandemic, including changes in dietary habits and physical activity.

As mentioned in the Introduction, the pandemic has impacted not only health but also the economy, which can have serious adverse effects on the mental health of families and children [27]. Economic stress has been found to worsen children’s mental health, mainly due to poor family relationships or quality of upbringing, which means that children from low-income families are less likely to have well-managed screen time during school closures and periods of social distancing [28]. Therefore, we propose that support and policies related to screen time also need to be carefully planned in consideration of economic status. For example, it is worth providing parental education related to screen time regulation, psychological counseling for parents, smart device addiction treatment for children, and alternative activities for children are considered first for low-income families.

The present study had several limitations. First, screen time was parent-reported and not verified against objective measures or self-reported by their children. In addition, the current study was not longitudinal; thus, we cannot ensure that children have changed their screen-usage patterns since COVID-19. However, it is expected that additional studies including the same participants and accumulated data from follow-up studies conducted since 2015 regarding screen time, can supplement this. The fact that information other than household income and changes in household income, such as parents’ educational background and employment status, and the number of children and other family members, was not included in the survey is also a limitation in interpreting the results. In future studies, relevant demographic information should be supplemented.

Although most previous studies examined children’s screen time by dividing them into two groups—low and high—based on median household income, this study provided more precision in income levels. Unlike many studies that have simply focused on income level, a strength of the present study may be its comparison of families whose household income decreased after the COVID-19 outbreak with those who did not, accounting for the economic situation created by COVID-19. Considering the collective results of this study, low-income families were more vulnerable in terms of screen time and mental health for both parents and children during COVID-19. Although these vulnerabilities were present before COVID-19, the results suggest that they increased when the economic situation worsened due to COVID-19. As the global pandemic continues, new guidelines that supplement existing guidelines for children’s screen time should be developed, and it is expected that policies and plans to support the mental health of vulnerable populations will be prepared. In addition, it would be helpful to provide psychological support, such as counseling services, to alleviate distress and guilt for parents experiencing high levels of depression and stress.

## Conclusion

During school closures due to COVID-19, children in low-income and declining income households engaged in more screen time than those in high-income families, which may also be closely related to mental health issues of the primary caregivers. Professional societies and policymakers should consider additional support for low-income families with children and provide guidelines for media use in establishing public mental health policies in prolonged pandemics.

## Data Availability

The datasets during and/or analyzed during the current study are available from the corresponding author on reasonable request.

## Abbreviations

BMI: Body Mass Index
COVID-19: Coronavirus Disease-2019
K-12: Kindergarten to grade 12
KRW: Korea Won
PC: Personal Computer
PHQ-9: Patient Health Questionnaire-9
SES: Socioeconomic Status
TV: Television
UNESCO: United Nations Educational, Scientific and Cultural Organization
